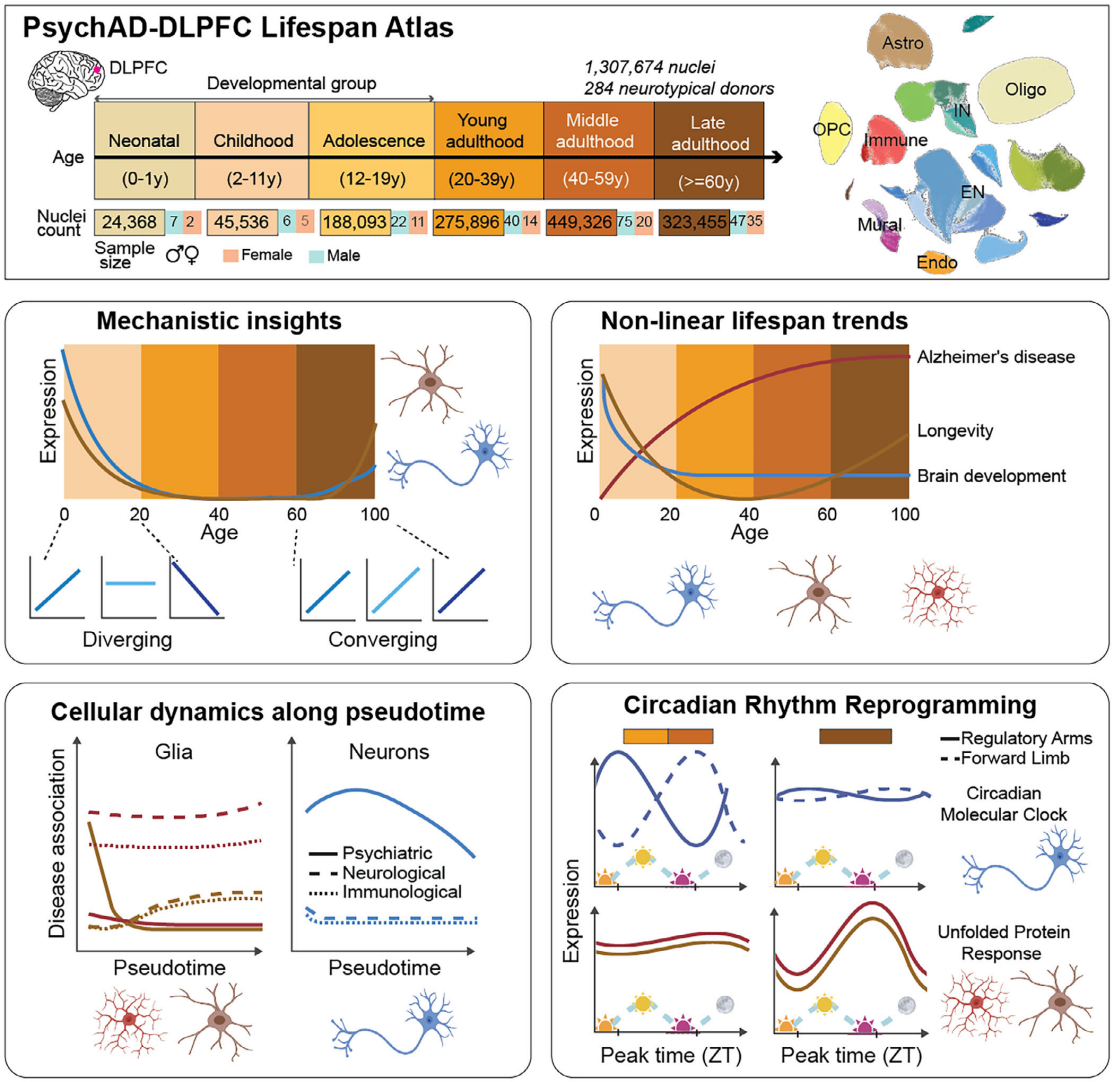# A single‐cell transcriptomic atlas of the prefrontal cortex across the human lifespan

**DOI:** 10.1002/alz70855_097914

**Published:** 2025-12-23

**Authors:** Kiran Girdhar

**Affiliations:** ^1^ Icahn School of Medicine at Mount Sinai, New York, NY, USA

## Abstract

**Background:**

Understanding the molecular mechanisms of human brain development, maintenance, and age‐related decline is crucial for interpreting the genetic architecture of developmental and late age related neurodegenerative diseases. While previous studies have successfully investigated age‐related transcriptomic changes within childhood (<20 yo) or adulthoods (>=20‐100 yo), no research to date has conducted a harmonized study spanning the entire lifespan to systematically create a cell‐specific reference aging trajectories of the human brain transcriptome.

**Method:**

To address these limitations, we conducted single‐nucleus RNA sequencing on the prefrontal cortex (PFC) region of 284 human postmortem controls spanning an age range of 0‐97 years. Our analytical approach was to track age specific changes across four distinct groups: childhood (0‐20 yo), young (21‐40 yo), middle (41‐60 yo) and late adulthood (>60 yo) across 26 major subclasses of diverse cells, including neuronal, glia, endothelial, and mural cells in the PFC, and integrated these changes with lifespan aging trajectories of every gene.

**Result:**

Our atlas revealed, during development, 2.9% and 1.7% of age‐related gene expression changes occurred in neuronal and glial subclasses, respectively. These changes stabilized after age 20 but significantly resurged after age 60, predominantly affecting glial subclasses compared to neurons. Quantification of the degree of sharing in transcriptomes across all age groups uncovered during aging, cellular stressors such as DNA damage, oxidative stress, and inflammatory responses become more pronounced, which activate shared pathways across different cell types, leading to a convergence of transcriptome signatures. We applied pseudotime approaches to identify dynamically expressed genes along lineage trajectories, and their disease associations, revealing critical windows of neurodevelopmental vulnerability and aging. Psychiatric disorder‐associated genes maintain high expression in neuronal lineages across the lifespan, indicating sustained involvement in brain function and diverse roles in development and maintenance. Conversely, genes linked to neurodegenerative diseases are highly expressed in microglia throughout the lifespan and prevail in oligodendrocyte lineages during aging.

**Conclusion:**

The resulting aging atlas provides: (a) a resource of the lifespan aging trajectory of every gene in 26 diverse subclasses of PFC and (b) valuable insights into the manifestation of common aging mechanisms across diverse subclasses.